# plethy: management of whole body plethysmography data in R

**DOI:** 10.1186/s12859-015-0547-7

**Published:** 2015-04-29

**Authors:** Daniel Bottomly, Beth Wilmot, Shannon K McWeeney

**Affiliations:** Oregon Clinical and Translational Research Institute, Oregon Health and Science University, 3181 SW Sam Jackson Park Rd97239, Portland, Oregon US

**Keywords:** Plethysmography, Respiration, Mouse, R

## Abstract

**Background:**

Characterization of respiratory phenotypes can enhance complex trait and genomic studies involving allergic/autoimmune and infectious diseases. Many aspects of respiration can be measured using devices known as plethysmographs that can measure thoracic movement. One such approach (the Buxco platform) performs unrestrained whole body plethysmography on mice which infers thoracic movements from pressure differences from the act of inhalation and exhalation. While proprietary software is available to perform basic statistical analysis as part of machine’s bundled software, it is desirable to be able to incorporate these analyses into high-throughput pipelines and integrate them with other data types, as well as leverage the wealth of analytic and visualization approaches provided by the R statistical computing environment.

**Results:**

This manuscript describes the plethy package which is an R/Bioconductor framework for pre-processing and analysis of plethysmography data with emphasis on larger scale longitudinal experiments. The plethy package was designed to facilitate quality control and exploratory data analysis. We provide a demonstration of the features of plethy using a dataset assessing the respiratory effects over time of SARS and Influenza infection in mice.

**Conclusion:**

The plethy package provides functionality for users to import, perform quality assessment and exploratory data analysis in a manner that allows interoperability with existing modelling tools. Our package is implemented in R and is freely available as part of the Bioconductor project http://www.bioconductor.org/packages/release/bioc/html/plethy.html.

**Electronic supplementary material:**

The online version of this article (doi:10.1186/s12859-015-0547-7) contains supplementary material, which is available to authorized users.

## Background

The ability to characterize phenotypes related to respiration is of vital importance to many areas of biomedicine ranging from pulmonary and infectious disease to drug discovery and biodefense. Although such devices can be applied clinically [1], plethysmographs designed for small animals provides an effective means to study phenotypes that can be expensive or impossible to carry out in humans. There are several variants of plethysmographs which differ in whether the animal is wholly contained within the chamber and whether they are restrained during the measurements [2]. For whole body plethysmographs [3] a common parameter of interest is enhanced pause (Penh), which can be considered a measure of airway resistance [4]. Some examples of the applications of these devices include studies in asthma [5], sleep apnea [6], cystic fibrosis [7], respiratory viral infection after radiation injury [8] as well as the assessment of host immune response to Respiratory Syncytial Virus after vaccination [9].

In order to easily analyze and share data from a large scale experiment utilizing unrestrained whole body plethysmographs we developed plethy. The plethy package is an R/SQLite based framework that enables the storage and retrieval of subsets of whole body plethysmography data for visualization and analysis. In addition, other measures that can be exported in a similar format, such as metabolic parameters, can also be utilized.

## Implementation

### Program design and data structures

The plethy package provides an infastructure for parsing, loading and querying plethysmography data utilizing SQLite databases. Currently, we provide parsing code designed to handle files exported from the Finepointe software suite bundled with the Buxco Whole Body Plethysmographer. An example of such a file is given as part of the example data (plethy.example.file) available at the github repository https://github.com/dbottomly/plethyData. These files are read into memory all at once or in chunks and loaded into an SQLite database. The user can control the number of lines read in at a given time. Although this can be useful for limiting the memory consumption of the program at the expense of runtime, there is a limit to its benefit as the data for several entire file sections are currently held in memory at a given time.

The use of SQLite provides several benefits in terms of simplicity and portability. These databases can be easily transfered amongst collaborators as stand alone files or as part of an encapsulating R package (see the ‘Parsing’ section of Additional file 1). This paradigm is one that has been utilized successfully by the Bioconductor project for distributing annotations [10]. In addition, the data contained within the database can be queried using SQLite’s own client or through drivers for most programming languages including R. The current schema for these databases is provided in Additional file 2.

The resulting database can be accessed conveniently through an S4 object oriented interface where a BuxcoDB object can be created and accessed through several defined methods. A listing of the available methods is provided in Additional file 3.

Retrieving data for analysis is done most conveniently through the retrieveData method. This method without any arguments will retrieve all the data and return it in a data.frame. This can take a lot of memory and will be a little slower than specifying the data subsets the user is interested in. By passing in character or numeric variables as arguments to the method (as shown in the ‘Data retrieval’ section of Additional file 1) smaller subsets of the data can be retrieved quickly and then be used for downstream statistical or graphical analysis. The form of the data.frame is described in Table 1.

**Table 1:** **Description of the fields in the** data.frame **returned by** retrieveData

**Column**	**Description**
Sample_name	Value corresponding to the ‘Subject’ column
P_time	Value corresponding to the ‘Time’ column in the form: ‘YYYY-MM-DD H:M:S’
Break_sec_start	The number of seconds elapsed since the beginning of the current run
Variable_Name	The current variable as determined by the columns after ‘Recording’
Bux_table_name	The name of the current table (e.g. WBPth)
Rec_exp_date	The factor provided by the experimentalist in the ‘Phase’ column
Break_number	The number of the current ‘break’ observed in the file as described above
Value	The value of the current variable

Multiple files can be imported to the database by simply running the parse.buxco function again and specifying the same database path. Alternatively, data.frames generated by retrieveData can be used to create a new database or be merged in with an existing plethy database using the dbImport method. This allows plethy to be extensible enough to be used in a collaborative multi-lab setting where data sharing is essential. Parsing and data retrieval is demonstrated in Additional file 1.

## Results and discussion

### Dataset

As a demonstration of the plethy package we used a plethysmography dataset derived from the longitudinal assessment of C57BL/6J mice after Influenza (Flu), Severe Acute Respiratory Syndrome Coronavirus (SARS) or mock infection (Menachery VD, Gralinski LE, Baric RS, Ferris MT: New metrics for evaluating respiratory pathogenesis, in preparation). This dataset is available as an R package at https://github.com/dbottomly/plethyData. Demonstration of these features and reproduction of the tables and figures is shown in Additional file 1 and Additional file 4. As is described in the ‘Parsing’ section of Additional file 1, the plethysmography files we work with are large and structured. Previously sections of these files would be loaded and manipulated in Excel by investigators, a process which was both tedious and error prone. The plethy package simplifies this process and enables efficient exploration and analysis of the data with only a handful of R functions. Assuming the data has been loaded into a database and a BuxcoDB object exists, we can first perform QA/QC of the data followed by exploratory data analysis and model fitting.

### Quality control

Currently our quality control procedures are centered around finding deviations from the expected number and duration of the measurements. For example, the expectation is that the Flu, SARS and mock infected animals were first allowed an acclimation period of 30 minutes in the chamber followed by 5 minutes of experimental readings. As shown in Additional file 1, these data can be labeled with their inferred run type (acclimation or experimental) and these labels can then be checked for similarity to the experimental design. If deviations are detected, the data can be re-labeled, keeping the old labels for provenance. The plethy package comes with procedures to provide basic checks of the measurement timestamps. One such check is performed internally while parsing and loading the data. This check emits a warning if observed experimental time intervals are greater than a specified tolerance. Similarly, the exact timepoint of an experiment can be computed from the measurement timestamps which are captured in the SQLite database. This enables automatic checking of the labeled experimental timepoint. In the case of the SARS, Flu and mock data, no such anomalies exist but we provide a simulated dataset where they do in the ‘Quality Control’ section in Additional file 1. These data can then be visualized using several built in methods as well as other visualization functionality in R.

### Visualization

**Figure 1:**
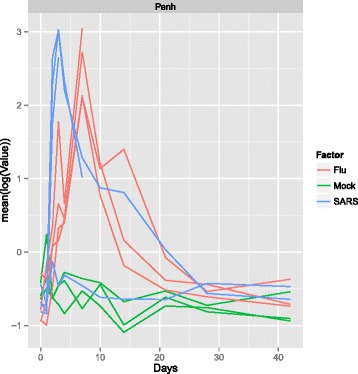
Line plot of the Penh phenotype. The mean log(Penh) response per animal per day is shown colored by the infection category (Flue, SARS or mock infection).

**Figure 2:**
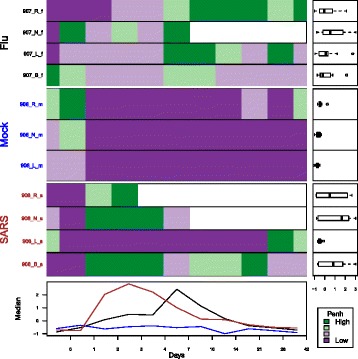
A multivariate time series plot of the Penh phenotype. A multivariate time series plot as defined in the plethy package combines a heatmap-like depiction of the variable distribution (middle; green is high, purple is low) for each sample along with a grouped trend line representing the median value colored by group (bottom). On the right is a boxplot summary of the phenotypic distribution.

Visualization is key for plethysmography data as many measurements can be produced for each animal for each time point. This results in a very rich multivariate experimental dataset which is also potentially longitudinal in nature. As our main use case is the change in respiration measurements over time, we provide two key plotting methods for longitudinal data: tsplot and mvtsplot. Arguably the most common way to visualize longitudinal data is using a simple line plot of the mean response per each animal and time point as is shown in Figure 1 for the Penh phenotype which can be produced using the tsplot method. Additionally, the mvtsplot method produces an adaptation of the multivariate time series plot [11] which combines a heatmap with boxplot-like summaries and a basic line plot to provide a detailed overview of a given experiment (Figure 2). Note that as is shown in both Figures 1 and 2, one of the SARS samples (908_L_s) appears to be anomalous compared to the others in terms of its Penh measurements and bears a greater similarity to the mock samples.

### Interface to modeling utilities

The simplest approach to analyzing longitudinal data is to summarize the data as a single value for each animal and then perform a T-test or analysis of variance, termed a ‘summary measures’ analysis [12]. We provide a summaryMeasures method which can compute a wide variety of summaries for each curve as demonstrated for animal ‘907_B_f’ below in Table 2. Also as mentioned above, retrieveData can provide the user with a data.frame and similarly data can also be returned in matrix form using the retrieveMatrix method. Importantly, either data.frames or matrices can serve as the input to almost all modelling functions in R. For example, we may be interested in assessing the effect of time and infection status on Penh values. As shown in Figures 1 and 2, the Penh response over time is non-linear showing a peak around Day 7 for Flu and 3 for SARS followed by a decline to roughly the level shown by the mock infected animals. An initial model for this might involve infection status and polynomial terms for time. Any such model should also account for the presence of many technical measurements for each sample. For the example code below we make the initial assumption that samples will differ only in terms of the intercept. Assuming bux.db refers to a BuxcoDB object we can fit a cursory mixed effects model [13] (only keeping the last 30 seconds of measurements) as:

**Table 2:** **Summary measures for animal ‘907_B_f’ for several Buxco whole body plethysmography phenotypes**

**Variable_name**	**Time_to_max**	**Max**	**Auc**	**Mean**
PAU	7.00	11.41	91.73	2.69
PEFb	7.00	9.43	266.88	6.49
Penh	7.00	11.84	74.00	2.23

As the goal of this section of the manuscript is only to highlight how plethy could be used in conjunction with other R packages we make no claims as to the efficacy of this particular model for a given analysis. As another example of utilizing the data retrieval functions to simplify analysis we can also perform classification. If more samples were accrued, we may also be interested in predicting Flu, SARS and Mock status given measurements from mutliple plethysmography outputs. One approach to this could be the use of linear discriminant analysis [14]. This could be carried out as below by first summarizing each sample by the mean phenotype for a given informative day, say Day 4, and running a procedure similar to below to train our model:

The predictions and assements of this model could then be carried out using utilities provided in one of many R packages.

## Conclusions

Our software provides a convenient framework for storage and retrieval of whole body plethysmography data in R. Currently we support import of data from the FinePointe software of Buxco, however the basic framework is amendable to data produced from other vendors. As the software is open source, such support can be requested via the Bioconductor mailing list or contributed via the github site (https://github.com/dbottomly/plethy) for the project. R in conjunction with SQLite allows efficient exploration and summarization of the data. Future releases of plethy will include additional built-in plotting and summarization tools.

## Availability and requirements

**Project name:** plethy**Project home page:**http://www.bioconductor.org/packages/release/bioc/html/plethy.html**Operating system(s):** Platform independent**Programming language:** R**Other requirements:** R >3.1.2, plethy ≥ 1.5.10**License:** GPL-3**Any restrictions to use by non-academics:** N/A

## Additional files

Additional file 1
**Sweave PDF vignette demonstrating manuscript analyses.** PDF demonstrating code from the plethy package including how to replicate the figures and tables using the example data.

Additional file 2
**The current database schema used by** plethy. A PNG file depicting the tables and relationships utilized by the parsing and data retrieval functions of plethy.

Additional file 3
**Table summarizing** plethy’s **API.** A CSV file listing the name, brief description and category of each of the currently defined functions/methods for plethy.

Additional file 4
**Rnw file corresponding to Additional file** 1. Rnw file used to produce the Additional file 1 PDF. The R code itself can be extracted using: R CMD Stangle plethy_supp.Rnw.
