# Supplementary material for: plethy: management of whole body plethysmography data in R
Source: BMC Bioinformatics. 2015 Apr 29;16(1):134. doi: 10.1186/s12859-015-0547-7 (PMC4434826; doi:10.1186/s12859-015-0547-7)
Supplement: Additional file 1 — Sweave PDF vignette demonstrating manuscript analyses. PDF demonstrating code from the plethy package including how to replicate the figures and tables using the example data. [file 12859_2015_547_MOESM1_ESM.pdf]

Additional File 1

Daniel Bottomly, Beth Wilmot and Shannon McWeeney

February 18, 2015

1 Installation

Below is the preferred way to install plethy and the example data used in this vignette. The only prerequisite is a version of plethy $\geq$ 1.5.10. Here we are using a development version of R as plethy 1.5.10 is currently part of the development version of Bioconductor. As Bioconductor packages are tied to specific R versions, installing the latest plethy in, say, R-3.1.2 is not supported (but will probably work).

```
> source( "http://bioconductor.org/biocLite.R" )
> biocLite( c( "plethy" , "devtools" ) )
> library( "devtools" )
> install_github( repo = "dbottomly/plethyData" )
>
```

2 Parsing

The plethy package provides code to parse files generated from the Finepointe software suite bundled with the Buxco Whole Body Plethysmographer which contain data of the form:

`Time,Subject,Phase,Recording,f,TVb,MVb,Penh,PAU,Rpef,Comp,PIFb,PEFb,Ti,Te,...`

Which is further organized into subsections containing either experimental or acclimation data based on the following pattern:

table.delim table name
header line
acclimation (ACC) for animal 1
burn.in.lines
acclimation (ACC) for animal 2
experimental readings (EXP) for animal 1
burn.in.lines
acclimation (ACC) for animal 3
experimental readings (EXP for animal 2

Where the **indicated** lines correspond to default parameters in the current implementation of the parse.buxco function. In plethy each header or **burn.in.lines** is termed a 'break' and is used to determine when the current run starts or ends. Definitions of the variables listed after the 'Recording' column are found in [Buxco Research Systems, 2005]. Unless the file format changes, these should not have to be modified. Below we provide an example of parsing the example data provided at https://github.com/dbottomly/plethyData.

```
> options( str = strOptions( vec.len=2 ) )
> library(  "xtable" )
> library ( "plethyData" )
```

```
> library ( "plethy" )
> file.name <- plethy.example.file ()
> chunk.size <- 10000
> db.name <-  file.path ( tempdir(), "bux_test.db" )
> bux.db <- parse.buxco ( file.name = file.name , chunk.size = chunk.size , db.name = db.name ,
+         verbose = FALSE )
```

In addition to stand-alone database, we can also create R packages containing the data. The benefit of these packages is that they can be easily distributed and provide a convenient means to access the data through `plethy's` R interface. Note that in this case `db.name` needs to follow R's package naming convention.

```
> db.name <-   "buxtest.db"
> parse.buxco ( file.name = file.name , chunk.size = chunk.size , db.name = db.name ,
+         verbose = FALSE,make.package=T)
> install.packages("buxtest.db", repos=NULL, type="source")
>
```

The `buxtest.db` package can then be interacted with similar to a standalone database:

```
> library(buxtest.db)
> samples(buxtest.db)
>
```

3 Adding Metadata

Note that at this point the data in the database consists of values directly parsed from the raw file. Adding additional metadata labels or numeric values can be done through the `addAnnotation` method by specifying the `BuxcoDB` object and a function returning the SQLite query. There are currently two functions that are defined, though users can define their own as well with some familiarity of the database structure. The first, `day.infer.query` computes the number of days past the first measurement for a given animal. The second `break.type.query` labels the measurements as 'ACC' for acclimation, 'EXP' for experimental, 'UNK' for unknown (where there is only one set of measurements for a given animal and given time point) or 'ERR' which likely represents an error in the parsing or query. Examples are shown below.

```
> addAnnotation ( bux.db, query = day.infer.query , index = FALSE )
[1] TRUE
> addAnnotation ( bux.db, query = break.type.query , index = FALSE )
[1] TRUE
```

The index argument specifies whether an index should be added to the annotation table. There should not really be any harm in adding them at each stage. However as the method tries to create a set of covering indices to maximize retrieval speed, we would recommend waiting until the last call to `addAnnotation` or until the execution of one of the helper functions which modify the annotation table (e.g. `add.labels.by.sample` or `adjust.labels`). These two functions construct such indexes automatically.

Metadata can also be imported to the database by specifying a `data.frame` to the `add.labels.by.sample` function defined by a 'samples' and optionally a 'phase' column (which we typically use to indicate the expected experimental time). Additional columns of this `data.frame` are then added to the annotations table. To demonstrate this we will add in the infection statuses for these mice as either 'SARS', 'Flu' or 'Mock' depending on the last character of the sample name. Note that in this example, `addAnnotation` could also be used as the names could be computed by an SQLite query.

```
> transl.table <- data.frame ( sample_status = c( "m" , "s" , "f" ),
+         Inf_Status = c( "Mock" , "SARS" , "Flu" ),
+         stringsAsFactors = F)
```

```
> use.dta <- data.frame( samples = samples ( bux.db ),
+         sample_status = sapply ( strsplit ( samples ( bux.db ) , "_"), "[", 3) ,
+         stringsAsFactors = F )
> merge.dta <- merge ( use.dta , transl.table , by = "sample_status" )
> merge.dta <- merge.dta [ , -which ( names ( merge.dta ) == "sample_status" ) ]
> add.labels.by.sample ( bux.db , merge.dta )
> annoLevels ( bux.db )
```

```
$Days
 [1]  0  1  2  3  4  7 10 14 21 28 42

$Break_type_label
[1] "ACC" "EXP" "UNK"

$Inf_Status
[1] "Flu"  "Mock" "SARS"
```

```
>
```

4 Data retrieval

We can extract the data using `retrieveData`. By default, everything in the database is retrieved in `data.frame` form. We can limit the retrieval to only subsets of the data by specifying constraints on several pre-defined variables parsed from the file: samples, variables, tables, phase and timepoint.

```
> data.1 <- retrieveData ( bux.db , samples = c( "906_L_m" , "908_R_s" ),
+         variables = c( "Penh" , "f" ))
> str( data.1 )
```

```
'data.frame':        28068 obs. of  11 variables:
 $ Sample_Name     : chr  "906_L_m" "906_L_m" ...
 $ P_Time          : chr  "2012-02-20 13:36:32" "2012-02-20 13:36:32" ...
 $ Break_sec_start : int  0 0 2 2 4 ...
 $ Variable_Name   : chr  "f" "Penh" ...
 $ Bux_table_Name  : chr  "WBPth" "WBPth" ...
 $ Rec_Exp_date    : chr  "Day 0" "Day 0" ...
 $ Break_number    : int  6 6 6 6 6 ...
 $ Days            : num  0 0 0 0 0 ...
 $ Break_type_label: chr  "ACC" "ACC" ...
 $ Inf_Status      : chr  "Mock" "Mock" ...
 $ Value           : num  496.545 0.681 ...
```

```
> table( data.1$Sample_Name , data.1$Variable_Name )
```

```
             f Penh
  906_L_m 9901 9901
  908_R_s 4133 4133
```

```
> data.2 <- retrieveData ( bux.db , samples = "906_L_m" , variables = "Penh" )
> with ( data.2, table ( Days, Break_type_label ) )
```

```
    Break_type_label
Days ACC EXP
  0  893 150
  1  886 140
  2  882 148
```

```
  3  868 147
  4  298 149
  7  888 150
  10 885 150
  14 891 145
  21 897 147
  28 889 148
  42   1 149

>
```

Additionally we can subset the data using the metadata columns defined through `addAnnotation` or `add.labels.by.sample`. For this we need to be able to type in the metadata column name and the constraint vector at the end of the `retrieveData statement`. The `annoCols` and `annoLevels` are convenient ways to determine which columns and values are available.

```
> annoCols ( bux.db )

[1] "Days"             "Break_type_label" "Inf_Status"

> annoLevels ( bux.db )

$Days
 [1]  0  1  2  3  4  7 10 14 21 28 42

$Break_type_label
[1] "ACC" "EXP" "UNK"

$Inf_Status
[1] "Flu"  "Mock" "SARS"

> data.3 <- retrieveData ( bux.db , samples = "906_L_m" , variables = "Penh" , Days = 0 )
> with ( data.3, table ( Days , Break_type_label ) )

    Break_type_label
Days ACC EXP
   0 893 150

> data.4 <- retrieveData( bux.db , samples = "906_L_m" , variables = "Penh" , Days = 0,
+         Break_type_label = 'EXP' )
> with ( data.4, table ( Days, Break_type_label ) )

    Break_type_label
Days EXP
   0 150
```

5 Quality Control

Although for a given run we typically carry out 900 acclimation measurements and 150 experimental measurements, there can be issues that arise which result in fewer measurements or even missing run types. One way to examine this is perform summaries of the 'Break_type_labels' inferred previously.

If discrepancies in terms of the labels are found, they can then be addressed by first extracting out information on the relevant entries, determining how they should be corrected and then adding in the new labels, keeping the old labels around for provenance. To demonstrate this we will first generate some additional data in an erroneous form using an internal utility function and create a temporary database.

```
> samples = c( NA , "sample_1" , NA , "sample_1" , "sample_2" , NA , "sample_3" ,
+          "sample_2" , NA , "sample_3", "sample_4" , NA , "sample_4" , "sample_2" ,
+          NA , "sample_2" , "sample_5" )
> count = c( NA , 900 , NA ,150 , 1 , NA , 900 , 28 , NA , 150 , 900 , NA , 150 , 900 ,
+         NA , 150 , 900 )
> measure_break = c( FALSE , FALSE , TRUE , FALSE , FALSE , TRUE , FALSE , FALSE ,
+         TRUE , FALSE , FALSE , TRUE , FALSE , FALSE , TRUE , FALSE , FALSE )
> table_break = c( TRUE , rep ( FALSE , length ( samples ) -1 ) )
> phase = rep ( "D1" , length ( samples ) )
> err.dta <- data.frame ( samples = samples , count = count ,
+         measure_break = measure_break,
+         table_break = table_break , phase = phase , stringsAsFactors=FALSE )
> sim.bux.lines <- plethy:::generate.sample.buxco ( err.dta )
> temp.file <- tempfile()
> temp.db.file <- tempfile()
> write ( sim.bux.lines, file = temp.file )
> test.bux.db <- parse.buxco ( file.name = temp.file , db.name = temp.db.file ,
+         chunk.size = 10000 , verbose = F )
> addAnnotation ( test.bux.db , query = day.infer.query , index = FALSE )

[1] TRUE

> addAnnotation ( test.bux.db , query = break.type.query , index = TRUE )

[1] TRUE

> type.summary <- proc.sanity ( test.bux.db )
> str ( type.summary )

List of 2
 $ time :'data.frame':        4 obs. of  3 variables:
  ..$ Break_type_label: chr [1:4] "ACC" "ERR" ...
  ..$ min_seconds     : int [1:4] 0 0 0 0
  ..$ max_seconds     : int [1:4] 1798 1798 298 1798
 $ count:'data.frame':        136 obs. of  5 variables:
  ..$ Sample_Name     : chr [1:136] "sample_1" "sample_1" ...
  ..$ Variable_Name   : chr [1:136] "Comp" "Comp" ...
  ..$ Days            : num [1:136] 0 0 0 0 0 ...
  ..$ Break_type_label: chr [1:136] "ACC" "EXP" ...
  ..$ num_entries     : int [1:136] 900 150 900 150 900 ...

> test.err.breaks <- get.err.breaks ( test.bux.db , max.exp.count = 150 ,
+         max.acc.count = 900 , vary.perc = .1 , label.val = "ERR" )
> str ( test.err.breaks )

'data.frame':        68 obs. of  7 variables:
 $ Sample_Name     : chr  "sample_2" "sample_2" ...
 $ Variable_Name   : chr  "Comp" "Comp" ...
 $ Rec_Exp_date    : chr  "D1" "D1" ...
 $ Break_number    : int  2 3 5 6 2 ...
 $ Break_type_label: chr  "ERR" "ERR" ...
 $ num_entries     : int  1 28 900 150 1 ...
 $ inferred_labs   : chr  "ERR" "ERR" ...

>
```

The 'test.err.breaks' `data.frame` indicates each sample, variable, phase and break that incurred an 'ERR' label in this example as well as the number of observations recorded. In addition, whether or not, the entry

can be reconciled is also reported as the 'inferred_labs' column. This is inferred by comparing the observed number of observations to the maximum number of expected experimental or acclimation observations. If the number of actual observations is greater than a 'vary.perc' percent decrease for either the acclimation or experimental units then it is labeled accordingly. The 'test.err.breaks' `data.frame` can be modified to address any discrepancies in the 'inferred_labs' column and then it can be used to adjust the metadata.

```
> adjust.labels ( test.bux.db , test.err.breaks )
> annoLevels ( test.bux.db )
```

```
$Days
[1] 0

$Break_type_label_orig
[1] "ACC" "ERR" "EXP" "UNK"

$Break_type_label
[1] "ACC" "ERR" "EXP" "UNK"
```

```
> data.6 <- retrieveData ( test.bux.db , samples = "sample_2" , variables = "Comp" ,
+         Break_type_label = "EXP" )
> str ( data.6 )
```

```
'data.frame':        150 obs. of  11 variables:
 $ Sample_Name          : chr  "sample_2" "sample_2" ...
 $ P_Time               : chr  "2015-02-18 21:20:22" "2015-02-18 21:20:24" ...
 $ Break_sec_start      : int  0 2 4 6 8 ...
 $ Variable_Name        : chr  "Comp" "Comp" ...
 $ Bux_table_Name       : chr  "WBPth" "WBPth" ...
 $ Rec_Exp_date         : chr  "D1" "D1" ...
 $ Break_number         : int  6 6 6 6 6 ...
 $ Days                 : num  0 0 0 0 0 ...
 $ Break_type_label_orig: chr  "ERR" "ERR" ...
 $ Break_type_label     : chr  "EXP" "EXP" ...
 $ Value                : num  0.194 1.275 ...
```

```
>
```

6 Plotting and Summary Utilities

Here we reproduce the figures and tables presented in the manuscript except for Table 1 which was compiled manually.

6.1 Time Series Plot (Figure 1)

```
> tsplot ( bux.db , variables = "Penh" , Break_type_label = "EXP" ,
+          exp.factor = "Inf_Status" )
>
```

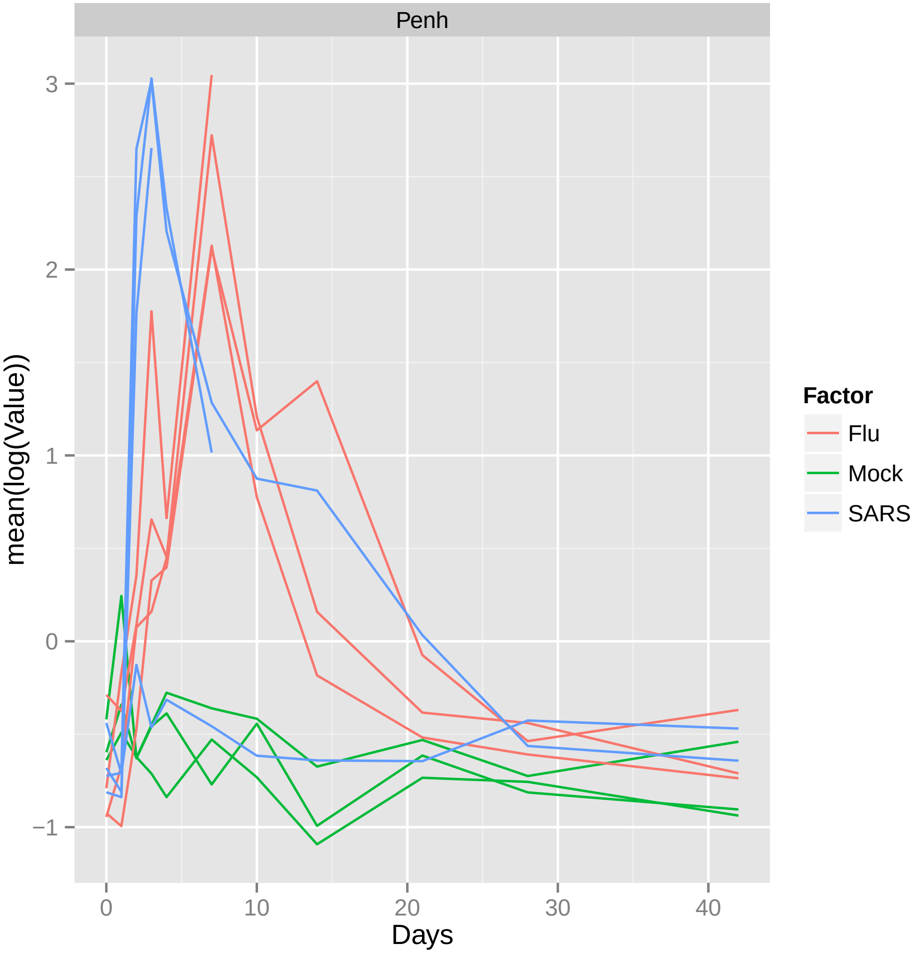

6.2 Multivariate Time Series Plot (Figure 2)

```
> mvtsplot ( bux.db , plot.value = "Penh" , Break_type_label = "EXP" ,
+          outer.group.name = "Inf_Status" ,
+          outer.cols = c( Flu = "black" , SARS = "brown" , Mock = "blue" ) )
>
```

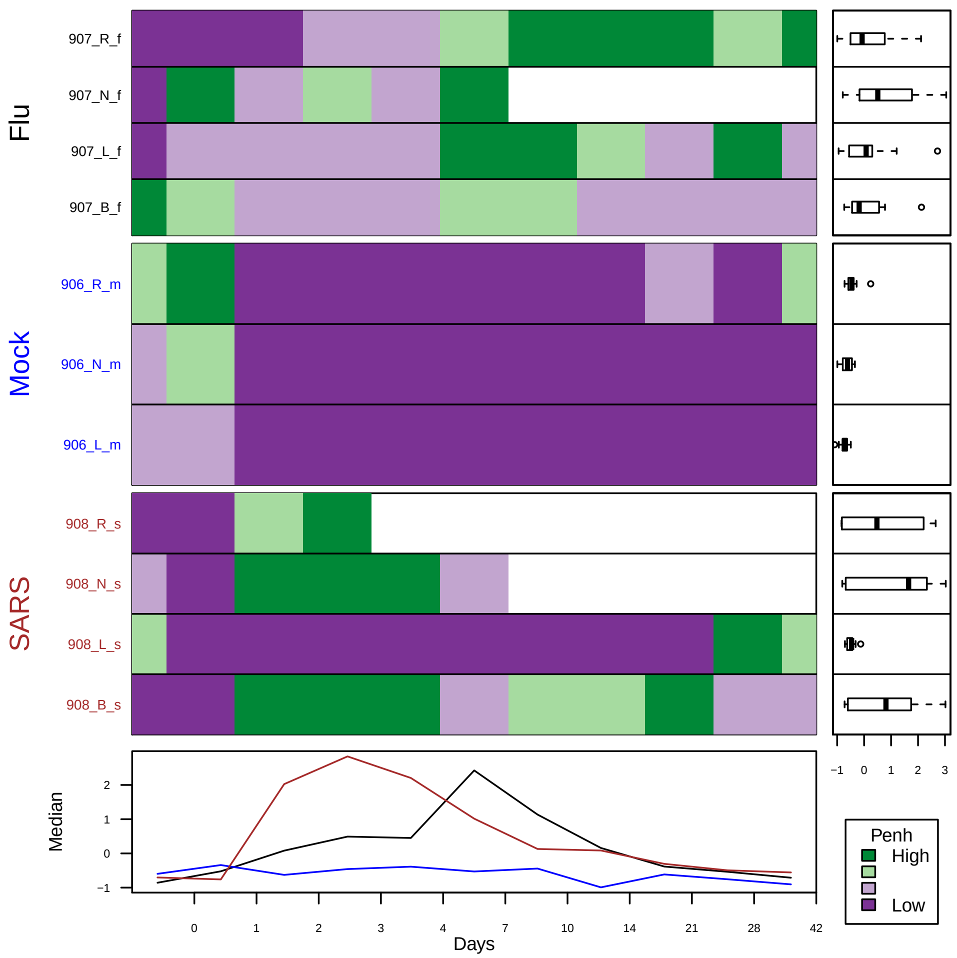

6.3 SummaryMeasures (Table 2)

```
> summaries <- summaryMeasures ( bux.db , samples = "907_B_f" ,
+         variables = c( "Penh" , "PAU" , "PEFb" ) , Break_type_label = "EXP" ,
+          tables = "WBPth" )
>
> use.summary <- summaries [ ,-which ( names ( summaries ) == "Sample_Name" ) ]
> names ( use.summary ) <- sub ( "\\.response" , "" , names ( use.summary ) )
> names ( use.summary ) <- gsub ( "\\." , "_" , names ( use.summary ) )
> print( xtable( use.summary,
+         caption = "Summary measures for animal '907\\_B\\_f' for several Buxco whole body plethysmogra
+         label = "table:sumTab" ,
+         align = rep("c", ncol(use.summary)+1)) ,
+          floating = T , table.placement = "h" , include.rownames = FALSE)

>
```

Variable_Name	time_to_max	max	auc	mean
PAU	7.00	11.41	91.73	2.69
PEFb	7.00	9.43	266.88	6.49
Penh	7.00	11.84	74.00	2.23

Table 1: Summary measures for animal '907_B_f' for several Buxco whole body plethysmography phenotypes

6.4 Model Fitting Examples

```
> library(nlme)
> penh.data <- retrieveData(bux.db,variables="Penh",
+         Break_type_label="EXP")
> sub.penh <- subset(penh.data, Break_sec_start > ( 298-30 ))
> lme( log( Value ) ~ Inf_Status + Days + I(Days^2) ,
+         random = ~1 | Sample_Name, data=sub.penh)
```

```
Linear mixed-effects model fit by REML
  Data: sub.penh
  Log-restricted-likelihood: -2255.997
  Fixed: log(Value) ~ Inf_Status + Days + I(Days^2)
   (Intercept) Inf_StatusMock Inf_StatusSARS           Days      I(Days^2)
    0.16509736    -0.83936077     0.34015448     0.03349208    -0.00122066

Random effects:
 Formula: ~1 | Sample_Name
        (Intercept) Residual
StdDev:   0.4502196  1.03181

Number of Observations: 1535
Number of Groups: 11
```

```
> library( MASS )
> bux.mat <- retrieveMatrix(bux.db,
+         Break_type_label="EXP",
+         Days=4,
+         variables=c("Penh", "PAU", "EF50"),
+         formula = Sample_Name + Inf_Status ~ Variable_Name)
> labels <- sapply(strsplit(rownames(bux.mat ),"_" ),"[",4)
> bux.data <- data.frame(labels=labels, bux.mat)
> trained.result <- lda(labels ~ EF50 + Penh + PAU,
+         data=bux.data)
>
```

```
> sessionInfo()
```

```
R Under development (unstable) (2015-02-03 r67717)
Platform: x86_64-apple-darwin12.6.0/x86_64 (64-bit)
Running under: OS X 10.8.5 (Mountain Lion)

locale:
[1] en_US.UTF-8/en_US.UTF-8/en_US.UTF-8/C/en_US.UTF-8/en_US.UTF-8

attached base packages:
[1] stats4    parallel  stats     graphics  grDevices utils     datasets
[8] methods   base
```

```
other attached packages:
[1] MASS_7.3-38         nlme_3.1-119        plethy_1.5.10
[4] S4Vectors_0.5.19    BiocGenerics_0.13.4 plethyData_0.99.0
[7] xtable_1.7-4

loaded via a namespace (and not attached):
 [1] graph_1.45.1       Rcpp_0.11.4        Streamer_1.13.1    IRanges_2.1.38
 [5] munsell_0.4.2      lattice_0.20-29    colorspace_1.2-4   stringr_0.6.2
 [9] plyr_1.8.1         tools_3.2.0        grid_3.2.0         Biobase_2.27.1
[13] gtable_0.1.2       DBI_0.3.1          RBGL_1.43.0        digest_0.6.8
[17] RColorBrewer_1.1-2 reshape2_1.4.1     ggplot2_1.0.0      RSQLite_1.0.0
[21] labeling_0.3       scales_0.2.4       proto_0.3-10
```

References

Buxco Research Systems. Respiration measurement in the whole body plethysmograph. Technical report, 2005. URL http://buxco.com/downloads/LomaskWBP.pdf.
