# Supplementary figures and images for: plethy: management of whole body plethysmography data in R
Source: BMC Bioinformatics. 2015 Apr 29;16(1):134. doi: 10.1186/s12859-015-0547-7 (PMC4434826; doi:10.1186/s12859-015-0547-7)

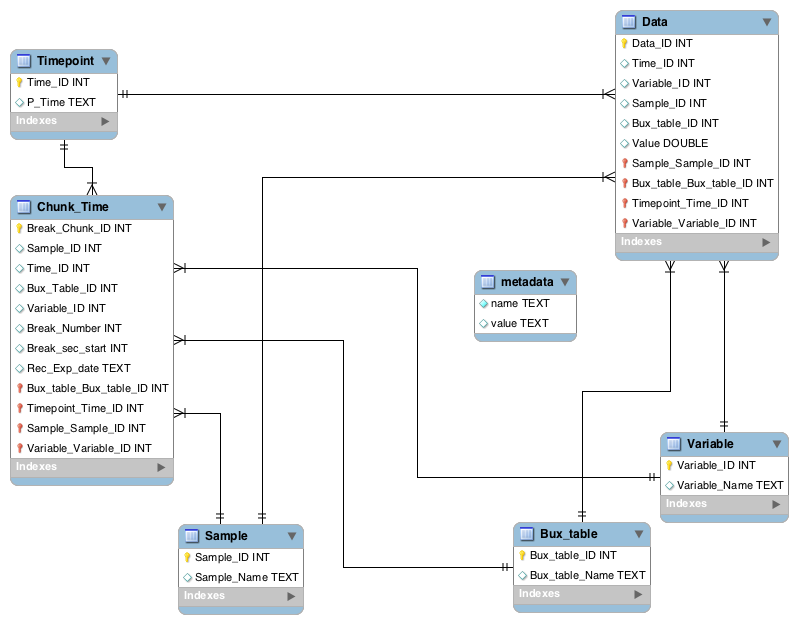

Supplement: Additional file 2 — The current database schema used by plethy. A PNG file depicting the tables and relationships utilized by the parsing and data retrieval functions of plethy. [file 12859_2015_547_MOESM2_ESM.png]
